# Counter-on-chip for bacterial cell quantification, growth, and live-dead estimations

**DOI:** 10.1038/s41598-023-51014-2

**Published:** 2024-01-08

**Authors:** K. M. Taufiqur Rahman, Nicholas C. Butzin

**Affiliations:** 1 Department of Biology and Microbiology, South Dakota State University, Brookings, SD 57006 USA; 2 Department of Chemistry, Biochemistry and Physics, South Dakota State University, Brookings, SD 57006 USA

**Keywords:** Imaging, Lab-on-a-chip, Microscopy, Nanobiotechnology, Microfluidics, Applied microbiology, Bacteria

## Abstract

Quantifying bacterial cell numbers is crucial for experimental assessment and reproducibility, but the current technologies have limitations. The commonly used colony forming units (CFU) method causes a time delay in determining the actual numbers. Manual microscope counts are often error-prone for submicron bacteria. Automated systems are costly, require specialized knowledge, and are erroneous when counting smaller bacteria. In this study, we took a different approach by constructing three sequential generations (G1, G2, and G3) of counter-on-chip that accurately and timely count small particles and/or bacterial cells. We employed 2-photon polymerization (2PP) fabrication technology; and optimized the printing and molding process to produce high-quality, reproducible, accurate, and efficient counters. Our straightforward and refined methodology has shown itself to be highly effective in fabricating structures, allowing for the rapid construction of polydimethylsiloxane (PDMS)-based microfluidic devices. The G1 comprises three counting chambers with a depth of 20 µm, which showed accurate counting of 1 µm and 5 µm microbeads. G2 and G3 have eight counting chambers with depths of 20 µm and 5 µm, respectively, and can quickly and precisely count *Escherichia coli* cells. These systems are reusable, accurate, and easy to use (compared to CFU/ml). The G3 device can give (1) accurate bacterial counts, (2) serve as a growth chamber for bacteria, and (3) allow for live/dead bacterial cell estimates using staining kits or growth assay activities (live imaging, cell tracking, and counting). We made these devices out of necessity; we know no device on the market that encompasses all these features.

## Introduction

Cell counting determines the number of cells in a sample volume^1^. Cell viability, cell-based assays, transformation, transfection, proliferation rate studies, and other assays require precise cell counts to ensure experiment standardization and test impact quantification^2,3^. The current technologies have limitations for bacterial cell counts, so we invested in making an alternative to them. The most widely-used and convenient method calculates colony forming units (CFU) by diluting cells on Petri plates, counting the colonies, and then back calculating how many CFU/ml were present in the liquid^4^. Though the CFU method is inexpensive, relatively accurate, and requires a minimal skill level, it has several disadvantages. It relies on the inaccurate assumption that each colony comes from a single bacterium. It displays only viable cells^4^, requires substantial processing time, and depends on accurate dilutions along with precise pipetting. Moreover, overcrowded and large clumps of cells can produce considerably imprecise results^4,5^. The ability of some bacteria to move (i.e., swim or spread) can make counting more challenging and often requires low colony numbers on the Petri dishes to do so. Moreover, slow-growing bacteria may take days to grow, and not all cells can grow in a lab setting on solid media^6^. Even with the fast-growing *E. coli*, researchers often wait overnight or longer to count colony numbers. As a result, researchers conducting time-dependent experiments need to estimate the cell number during the investigation and then wait to calculate the actual number of bacteria used after tallying the CFUs. For example, in competition experiments, the ratio (e.g., 1:1, 1:10, etc.) of competing microbes is critical to understanding the relationships between cell types. However, researchers using the CFU method must estimate this ratio while setting up experiments.

In research and healthcare labs, accurate bacterial counts are indispensable to identifying potential health disorders and providing guidance and treatment without delay. For example, urinary tract infection (UTI) is commonly caused by bacterial infections. They are currently diagnosed in the USA using the widely accepted cutoffs of 10^5^ CFU/ml for adults or older children^7,8^, and ≥ 50,000 CFU/ml for infants 2–24 months^9,10^. Therefore, inaccurate counting increases the likelihood of underdiagnosis and impedes the development of an appropriate treatment plan. Hence, a precise bacterial count is necessary for accurate UTI diagnosis to prevent complications and recurrent infections^9,10^. Though the CFU/ml method is currently used to calculate cutoffs, this method does not readily consider persister cells and viable but not culturable (VBNC) cells.

Persisters are a subpopulation of tolerant cells, which can sustain longer stress than the slow-growing dying cells by entering a metabolically altered state where they do not divide^11–15^. It has been predicted that VBNCs cannot grow on Petri plates but can only grow in liquid, and that VBNCs may be related to antibiotic-resistant development^16–18^. Currently, there is a furious debate if VBNCs are actually persisters (persisters were identified about 40 years before VBNCs^19,20^ and share phenotypes), are simply dying cells, or if VBNCs even exist^21–25^. Cell counting accuracy has been a stubborn challenge as the established methods can be highly dependent on a multitude of factors, such as precise dilutions and pipetting, imaging technology, and appropriate reference materials. Thus, a convenient and timely counting approach may be a helpful tool for studying how bacteria survive stresses such as antibiotics. This problem was one of our motivations for developing the devices described in this article. We could not match others reported VBNC numbers, which led us to consider the inaccuracy of the current cell counting methods as a cause.

A common but inaccurate practice is to rely on Optical Density (OD) to estimate cell numbers; there is no linear relationship between cell count and OD, and ODs do not produce a direct measure of cell count^26–28^. Moreover, OD measurements show inaccurate results for viable bacteria count due to the influence of various stress factors (e.g., temperature, pH)^29^ because cell size and shape are influenced by stress. In layperson's terms, the size, shape, number of dead cells, and internal and external bacterial components can affect the OD measurement. In addition, the environment can alter these bacterial attributes, resulting in inaccurate OD estimates. A significant drawback of using ODs as a proxy for cell growth is additional calibration is required for higher throughput assays. For example, high-precision microplate reader assays require recalibrated microbeads in each well in each plate (often 96–284 wells per plate) to calibrate between wells^26,27^. Accurate cell numbers in microplates are often validated using CFU/ml for experiments, leading to additional work and time. Getting an accurate count of bacterial communities (e.g., biofilms) can be challenging. The population may contain multiple species of bacteria or the same bacteria but with different attributes (e.g., in biofilms, bacteria of the same species often differentiate, so they have different sizes, shapes, etc.).

Diagnostic labs, university labs, and pharmaceutical and biotechnology companies typically use manual (e.g., hemocytometers) and automated (e.g., Coulter counters)^30^ cell counting methods to quantify the number of cells, particles, and microorganisms. Hemocytometers are well-known manual counters used for over a century in a microscope to count blood cells, particles, and some microscopic organisms^31^. Currently, the Petroff-Hausser (PH) counting chamber (Hausser Scientific) is used to quantify bacteria and sperm, as it offers a series of cell depths (10, 20, and 40 microns) suitable for smaller cells^32,33^. The PH counter requires a higher skill level than the CFU/ml method, is time-consuming, and has low throughput. PH has a counting discrepancy of 20–30% (Hausser Scientific) when averaging the counts of 3 biological replicates. It is important to note that without doing the 3 replicates, it is wildly inaccurate. This inaccuracy may account for over- or under-estimating the cell number within a culture. It is only accurate depending on the number of cells loaded and is only precise at around 1 × 10^6^ cells/ml^1,34^. If the PH protocol is not precisely followed as described by the manufacturer, these errors can be significant. For example, based on our own results with *E. coli*, the number of cells can be overestimated by about twofold, suggesting there are equivalent numbers of VBNCs as CFU/ml. This would be consistent with previous literature where equal counts of VBNCs were counted per non-VBNCs in exponential and stationary phase^35–37^. However, if the protocol is followed precisely, we see no evidence of VBNCs in the exponential phase^38^. Though strictly following the manufacturer’s protocol increases the time per experiment dramatically (due to the extra replicates, and it may require additional steps to concentrate or dilute cells to get within the PH proper range), it is required for accuracy.

Automated cell counters (e.g., Coulter counters, flow cytometry) offer a higher throughput option compared to PH by measuring the volume of each sample through a fluid-based control system. The automated Cedex HiRes Analyzer and Cellometer counter ranges are approximately 3.13 × 10^5^–1.0 × 10^7^ cells/ml ^39^, and 1.0 × 10^5^–1.0 × 10^6^ cells/ml^40^. However, the range is not specifically stated for small bacteria counts. Notably, this intricate process requires expertise for accurate results and can be expensive. Microscopic observation and counting of submicron-sized bacteria using the PH counter or automated cell counters can be challenging, but many industrial, environmental, and medical-relevant bacteria are quite small: *E. coli* (diameter ~ 1.0 μm)^41^, *Lactococcus lactis* (diameter ~ 0.75–0.95 μm)^42^, *Prochlorococcus* (diameter 0.5–0.7 μm)^43^, and Mycoplasma (diameter ~ 0.2 to 0.4 μm)^42^. The smaller the cell, the more difficult to count, and the larger the chamber depth, the harder it is to count cells. A perfect auto-focusing control system is essential to obtain high-quality images^44^. Because live cells are highly motile and stay in different *x*–*y* planes^45^, counting them in a counting chamber (^**↑**^depth ^**↑**^*x–y* planes) can be challenging. An advanced microscopic focal plane imaging system (i.e., Z-stack) can identify the focal region of a cell^44^; nevertheless, it requires extensive imaging and further image stacking, resulting in non-specific cross-over and background noise^46^. Getting high-quality Z-stacks of bacteria takes significant expertise, expensive instruments, and it is not a nontrivial undertaking, especially with extremely small bacterial cells. If your goal is to know the number of cells quickly (i.e., for competition experiments) and easily, these systems may not be ideal, especially for fast-grown microbes. Furthermore, some bacteria grow exceptionally slowly (e.g., nitrogen-fixing Rhizobia have a doubling time of ~ 6 h^47^ and grow to low density). Low density cell cultures often need to be concentrated before loading them into the automated cell counters or PH, which adds another potential source of error and additional time to each experiment.

An alternative to the currently used instruments for cell counting would be to design a new type of intricate microfluidic device. Addressing the challenges of fabricating intricate, high-precision micro/nanostructures, a groundbreaking 2-photon polymerization (2PP) technology has shown great potential. By leveraging this technology, it becomes possible to fabricate arbitrary three-dimensional (3D) structures using various materials, achieving resolutions beyond the diffraction limit (as small as 100 nm)^48–50^. This advancement dramatically enhances the dimensional accuracy, shape fidelity, and surface smoothness of the resulting structures, thereby influencing the overall processing accuracy. Unlike traditional photolithography systems, the 2PP employs a lay-by-lay method using femtosecond pulse lasers^48,49^, enabling the rapid printing of complex structures, facilitating easy modifications, and capable of in-chip printing inside a sealed channel. This fabrication process shifts the paradigm of establishing a dynamic microfluidic device, allowing for precise control of in vitro experiments, and enabling real-time investigation under microscopy^49^, such as 2PP-based microfluidic cell sorter^51^.

In this study, we used a 2PP 3D printing system (Nanoscribe GmbH) to fabricate novel counter-on-chip systems. We optimized the 2PP-fabrication and polydimethylsiloxane (PDMS)-based chip construction process to ensure reproducibility and efficiency. Our simplified and refined methodology demonstrated excellent accuracy and precision, enabling the rapid construction of PDMS-based chips. We describe in detail this process (from 2PP fabrication to PDMS-chip construction) so others can easily repeat it, and because there is a lack of clarity in the literature on making such devices. We specifically describe in detail how we have overcome the 3D printed structure shedding off caused by PDMS curing because this was a major hurdle we faced.

We developed three generations (G1, G2, and G3) of counter-on-chip that can precisely count microbeads (1 µm and 5 µm) and bacterial cells. As a *proof of principle*, we used the G2 and G3 counters to count *E. coli* cells (~ 1 µm diameter). We further describe that our systems offer more convenient features than the traditional CFU/ml method. Of all, the G3 device showed better efficiency due to its smaller chamber size and reduced depth (5 µm). G3 can provide accurate bacterial counts, is used as a growth chamber for bacteria, and enables the estimation of live/dead bacterial cells using staining kits or growth assay activities, including live imaging, cell tracking, and cell counting.

## Results and discussion

### Counter-on-chip fabrication

Previous researchers have fabricated structures using the 2PP system; however, we simplify the method and provide comprehensive details on choosing photo-resin, objective, and other parameter settings, which are required to reproduce our counter-on-chip devices. Our method relies on the recent work of others, trial-and-error tactics, and empirical validation. The current study modified the process for enhancing the adhesion properties of a silicon substrate (SS), enabling the construction of a reusable, efficient, and user-friendly PDMS-based chip. We employed a 2PP system to generate a simple planar structure, considering the potential challenges of printing and fabricating intricate micro-/nanostructures in future studies, as high-precision structure presumably needs to deal with sub-micron size bacteria (e.g., Mycoplasma). The current refined methods using the 2PP system showed great potential to microfabricate our structures rapidly. This advanced technology has facilitated the development of accurate, reusable, and relatively cost-effective PDMS chips. We decided not to use conventional photolithography to develop this counter because the 2PP system is accurate, easy to use, and quick to produce a device. In our experience, traditional photolithography accuracy below 5 microns is problematic across an entire chip (we need all chambers to have highly accurate heights and widths for the counts to be valid), and to get these accurate structures over an entire chip often takes several attempts. In addition, the 2PP system does not require a cleanroom, or resins modification (e.g., mixing SU-8 resins) and is easy to operate.

**Figure 1:**
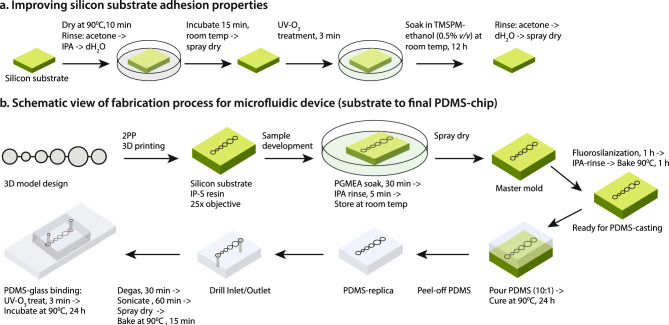
(**a**) Method for enhancing the adhesive qualities of silicon substrates. The silicon substrate is non-transparent and highly efficient in fabricating PDMS microfluidic chips. TMSPM, a coupling silane agent, increases the adhesion of the silicon substrate, resulting in better polymerization when a 2PP 3D printing system is used. (**b**) The fabrication process of a microfluidic counter, from the substrate to the final PDMS chip. The 2PP system used a CAD model to print a 3D structure. PGMEA dissolved unpolymerized resin to produce the structure, while IPA removed excess resin. Flurosilanization helps to isolate cured PDMS from the silicon substrate. Both the inlet and outlet were drilled using a biopsy puncher. Finally, UV-O3 treatment was performed to bind PDMS to a glass slide. PDMS: Polydimethylsiloxane, 2PP: Two-photon polymerization, IPA: Isopropyl alcohol, UV-O_3_: Ultraviolet ozone, TMSPM: 3-(Trimethoxysilyl)-propyl methacrylate, PGMEA: Propylene glycol monomethyl ether acetate.

The polymerized resin used with the 2PP system may create structural instability, leading to sagging or ultimately collapse of the structure if there is less adhesion between the substrate and resin. Therefore, photoresist and substrate selection are crucial. For maintaining spatial resolution and high adhesion, a negative-tone acrylate-based photoresist such as IP-series resists (here we used IP-S) and silicon substrate (SS) (Nanoscribe GmbH) could be an excellent choice^50^. However, we also tested other resins including IP-Q, IP-PDMS, IP-Visio; and substates like ITO-coated and fused silica. Here, we demonstrated an improved method for enhancing SS adhesion properties known as silanization (Fig. 1a). Silanization increases the adhesion between SS and polymeric microarchitectures^52^. After silanization, we kept the SS in a Petri plate to avoid contamination such as dust. The silanized SS performed well immediately and a few days later. However, we do not recommend waiting too long to print on the silanized SS. PDMS generally does not stick well to the substrate due to its low surface energy^53^. Silanization makes a strong bond between SS and PDMS, which may result in structure shedding off from SS during PDMS demolding. To avoid shedding, we employed 10 μL of anti-adhesion silanizing agent (1H, 1H, 2H, 2H-Perfluorooctyltriethoxysilane) (FOTS) immediately after spray drying (Fig. 1b). This fluorosilanization process reduces the adhesion between PDMS and SS. This procedure resulted in the smooth separation of the PDMS replica from the mold after curing. Under the vacuum, FOTS evaporates and modifies the surface chemistry by generating a tiny monolayer on SS, making it super-hydrophobic and preventing PDMS from adhering to SS^54,55^. It was recently reported that the fluorosilanization process should repeat after 10 subsequent uses of the silicon wafer as a master mold^56^. However, we tested the same fluorosilanized master mold more than 30 times, and yet no structure shedding was observed after PDMS curing. This demonstrates the accuracy and consistency of our refined process.

PDMS is slightly hydrophobic, so it is necessary to make the surface active or increase surface roughness to bind to the glass substrate covalently. Some commonly used bond-strength methods are oxygen plasma treatment^57–61^, ultraviolet-ozone (UV-O_3_)^61–63^, or corona treatment^61^. Collectively, all methods have some advantages and disadvantages discussed in Ref^61^. We used UV-O_3_, which was used to clean semiconductor material as early as 1972^64^. UV-O_3_ cleaning significantly reduces organic residues or contaminants^65^ and activates the surface without affecting surface structure^66^. In addition, this cleaning method permits PDMS to create a hydroxyl group on its surface, leading to irreversible bonding between the PDMS and glass substrate^67^. In this study, we applied 3 min. of UV-O_3_ treatment for PDMS surface activation and further bonding to the glass substrate by baking at 90 °C for 24 h (Fig. 1b).

It is noteworthy to mention that baking both the PDMS mold and glass substrate at 90 °C for 20 min. before UV-O_3_ treatment enhanced the binding strength.

### Structure characterization

**Figure 2:**
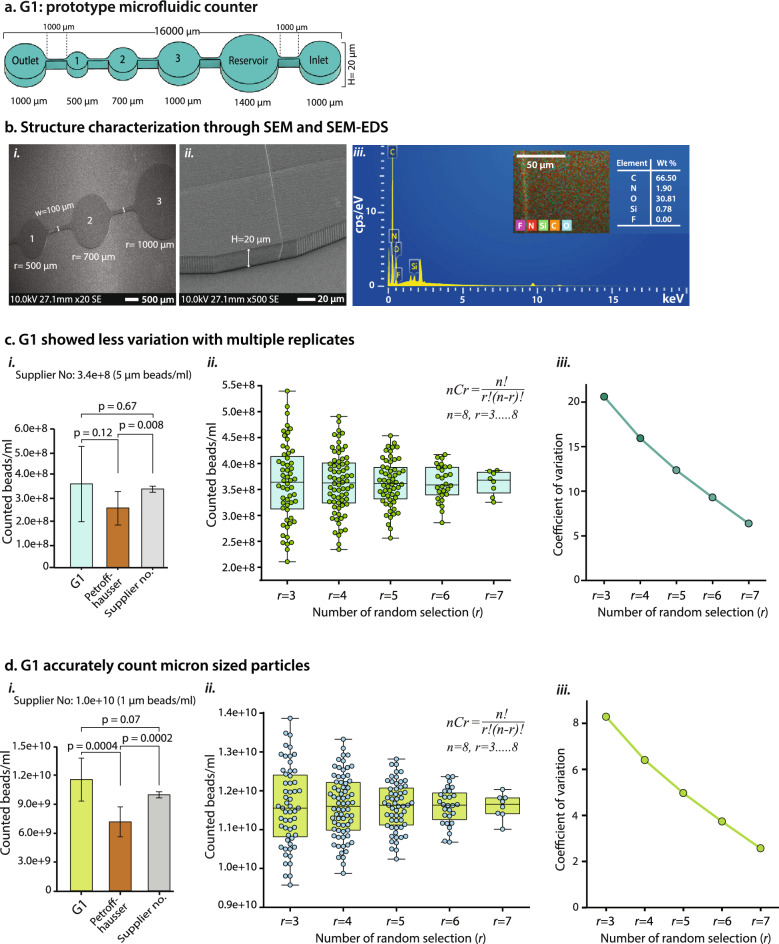
(**a**) G1-prototype of a microfluidic cell counter consists of three chambers, an inlet, an outlet, and a reservoir with a depth of 20 µm (H = height/depth). All chamber size is depicted in radius (r). (**b**) Overview of SEM and SEM–EDS: (**i**) The SEM image validated the accuracy of the chamber and structure dimension. (**ii**) A tilted angle SEM image showed the depth of the G1 counter. (**iii**) SEM–EDS showed the elemental properties of the G1 counter. Five different atomic compositions were analyzed. Florin (F) was added as a control. (**c**) Comparative analysis of G1, Petroff-Hausser, and supplier data with 5 µm microbeads: (**i**) G1 showed no significant difference to PH and supplier count (p = 0.67, n = 8), whereas PH and supplier count showed significant count differences (*p* = 0.008, n = 8). (**ii**) The combination math approach illustrated that multiple replicates lead to less variation; *r* = number of random selections, n = number of replicates (See Methods). (**iii**) More replicates have a lower coefficient of variation. (**d**) Micron-sized beads (1 µm) can be accurately counted with G1. (**i**) G1 showed no significant variation with supplier count (*p* = 0.07, n = 8) but a significant difference with PH (*p* = 0.0004, n = 8), whereas PH and supplier count showed significant count differences (*p* = 0.0002, n = 8). (**ii**) The more the replicate numbers, the less the count variation and outliers. (**iii**) Increasing the number of replications reduces the coefficient of variation. w: Channel width, n: Number of replicates, SEM: Scanning electron microscope, SEM–EDS: SEM-energy dispersive X-ray spectroscopy.

**Figure 3:**
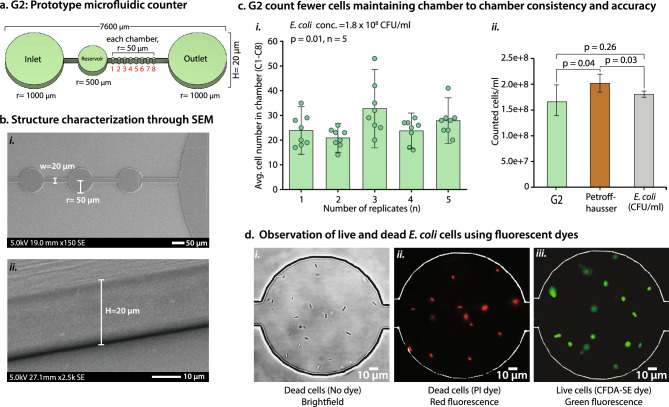
(**a**) G2-prototype of a microfluidic cell counter. G2 has eight equal-size chambers (each chamber radius, r = 50 µm) with an inlet, an outlet, and a reservoir with a depth/height (H) of 20 µm. (**b**) SEM images validate the G2 structure dimensions. (**i**) SEM confirmed the size accuracy of the channels and counting chambers. (**ii**) The titled angle SEM image depicted the height/depth (20 µm) of the G2 counter. (**c**) G2 counts fewer cells with high accuracy. (**i**) G2 contains an average of 20–35 cells in five replicates and has significant count variation (p = 0.01, n = 5). (**ii**) G2 total count showed no significant count difference with CFU counts (*p* = 0.26, n = 5). (**d**) Identification of live and dead cells using live/dead staining kits. (**i**) Microscopic brightfield imaging (1000× magnification) confirms the presence of *E. coli* cells. (**ii**) PI shows red fluorescence on dead cells (excitation/emission of 493/636 nm). (**iii**) On live cells, CFDA-SE exhibits green fluorescence (excitation/emission of 492/517 nm). n: Number of replicates, w: Channel width, PI: Propidium iodide, CFDA-SE: Carboxyfluorescein diacetate succinimidyl ester.

**Figure 4:**
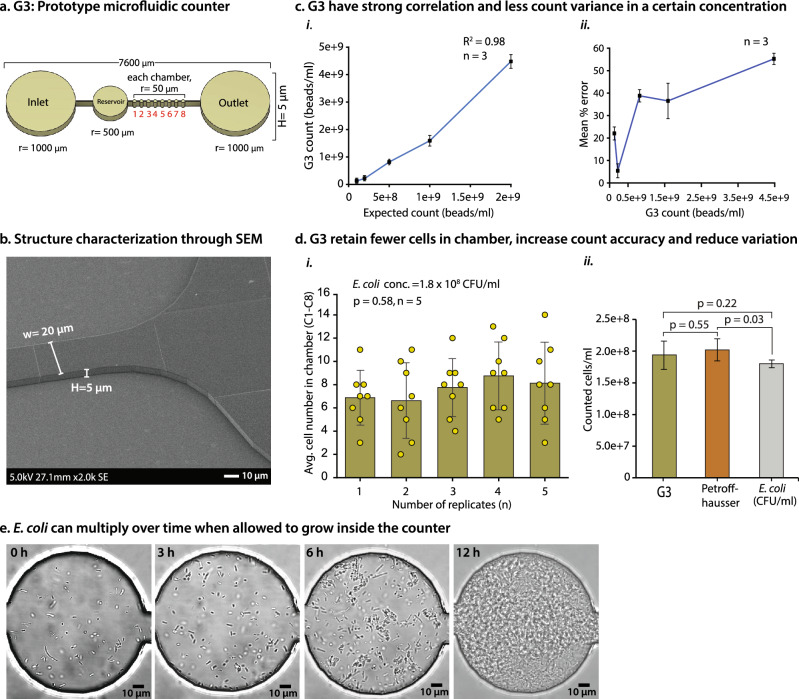
G3-prototype of a microfluidic cell counter. (**a**) G3 consists of eight equal-size chambers (radius, r = 50 µm) with an inlet, an outlet, and a reservoir with a depth/height (H) of 5 µm. (**b**) SEM images validate the depth (H = 5 µm) and channel width (w = 20 µm) of the G3 structure. (**c**) G3 showed a strong correlation and less variation with estimated counts compared to predicted counts. (**i**) Five different concentrations (1.0 × 10^8^, 2.0 × 10^8^, 5.0 × 10^8^, 1.0 × 10^9^, and 2.0 × 10^9^ beads/ml) of 1 μm beads were compared to the G3. For each concentration, n = 3; R^2^ = 0.98. (**ii**) Reduced concentrations lead to better counting accuracy and fewer variations. Here, 2.0 × 10^8^ beads/ml showed only a 5.4% count difference, whereas higher concentrations such as 2.0 × 10^9^ beads/ml showed a 55.3% difference from the expected counts (n = 3). (**d**) G3 contains very few cells in each chamber, which reduces variation and enhances count accuracy. (**i**) The average cell numbers (n = 5) were ≤ 10 with no statistically significant variation (*p* = 0.58, n = 5) in chamber-to-chamber counting. (**ii**) G3 shows no significant difference with the CFU/ml (*p* = 0.22, n = 5) and PH count (*p* = 0.55, n = 5). **e.** Time-lapse images show *E. coli* growing in the G3 counter using LB media. n: Number of replicates.

The structure accuracy and elemental properties were validated using scanning electron microscopy (SEM) (Figs. 2b i-ii, 3b i-ii, 4b) and SEM–EDS (energy dispersive X-ray spectrometer) analysis (Fig. 2b iii). All three-generation counter’s SEM images showed the expected chamber dimension, channel width, and overall depth (Figs. 2b i-ii, 3b i-ii, 4b). SEM showed the precise depth (20 μm) of the G1 (Fig. 2b ii) and G2 counter (Fig. 3b ii). We kept these counters depth similar to the depth (20 μm) of Petroff-Hausser (PH) because PH is currently routinely used to count bacterial cells^33^. In general, bacteria move a lot and stay in different phases, leading to difficulty in precision imaging and counting. To address these issues, we introduced the G3 counter with a lower depth (5 μm) (Fig. 4b), which can control high cell motility and reduce cells to stay in many phases. EDS spectrum of G1 (chamber 1) showed the elemental composition of the structure as expected: 66.5 wt% C, 1.9 wt% N, 30.81 wt% O, and 0.78 wt% Si and 0% F (as a control) (Fig. 2b iii). G2 and G3 had similar atomic compositions as G1 (data not shown). The EDS spectrum showed a homogeneous distribution of each element and was not affected by any other elements that could defect the structure. These results confirmed that the master mold was ready for PDMS molding. We used the acrylate-based IP-S photoresists (Nanoscribe GmbH), whose atomic composition is mainly carbon, hydrogen, and oxygen^68,69^.

### Quantification of microbeads using G1 counter

The G1 device (Fig. 2a) was used for initial process optimization such as sample preparation, sample loading, washing, and microscopic imaging. G1 contains three distinct-sized counting chambers, each with a radius (r) of 500, 700, and 1,000 μm, respectively (Fig. 2b i). The chamber-to-chamber volume was increased two-fold. We designed 3 distinct size counting chambers to identify whether counting accuracy is affected while increasing the volume or cell concentration. The reservoir (r = 1400 μm) was used to control the initial pressure or flow turbulence. The 100 μm channel width helps to slowly move the cells/particles in the main counting chamber.

G1 and PH counters were first compared using 5 µm (3.4 × 10^8^ beads/ml) and then 1 µm (1.0 × 10^10^ beads/ml) green fluorescence microbeads. The PH counter was used to set a reference point to analyze the variation of the G1 and validate the supplier counts; the manufacturer description 20–30% count discrepancy is expected in PH. Our first-generation G1 counter and the supplier’s count were quite similar. The PH count for 5 µm and 1 µm beads varied by 24.2% (Fig. 2c i, Supplementary Fig. S2i) and 28% (Fig. 2d i*,* Supplementary Fig. S2ii), respectively, which are within the manufacturer's expected error range. For both sizes of beads, the count difference was statistically significant (*p* = 0.008 and *p* = 0.0002) compared to the supplier counts. In the G1 device, we used three chambers in a row to count the 5 µm beads, and two consecutive chambers (1 and 2) to count the 1 µm beads to reduce challenges associated with imaging and counting. The 5 µm beads count in G1 had no statistically significant difference compared to the supplier count (*p* = 0.67) and PH count (*p* = 0.12). The number of counted beads varied by 6.6% (mean percent error) in the G1 device compared to the supplier counts (Fig. 2c i*,* Supplementary Fig. S2i). On the other hand, 1 µm bead count showed no significant count difference compared to the supplier counts (*p* = 0.07) but showed statistically significant variation compared to PH (*p* = 0.0004). The number of counted beads varied by 16% (mean percent error) in the G1 device compared to the supplier counts (Fig. 2d i*,* Supplementary Fig. S2ii). These results show that the G1 device is quite accurate compared to PH and the supplier’s counts. Increasing the number of replicates should increase accuracy, but we stopped at 8 replicates to minimize the workload and time for each run. We further investigated the possible number of replicates using combination math approaches for both 1 µm (Fig. 2c ii) and 5 µm beads (Fig. 2d ii) (see Methods). Increasing the replicate numbers reduces outliers and variations; the coefficient of variation was also decreased in both cases (Fig. 2c iii,d iii).

Although the G1 counter demonstrated relatively satisfactory results, it highlighted certain drawbacks, including the need for laborious imaging and data processing. Notably, the large chamber size did not significantly impact counting accuracy compared to PH and supplier counts but made imaging and data processing tedious and time-consuming. G1 can be efficiently used for counting bigger cells/particles (≥ 5 µm) with 100 × or 1,000 × magnification with a microscope setting. However, the detection of smaller-sized bacteria required higher magnification. In G1, chamber 3 is 1000 µm in radius, which means many images would need to be processed at 1000 magnification as our in-house inverted microscopy field of view (FoV; the size that can be imaged at once) is ~ 180 µm based on its radius. Our next goal was to produce a better device that required fewer images because this should reduce counting errors that may occur from combining images and decrease the time in counting. We achieved this goal by developing G2 and G3 microfluidic cell counters.

### *E. coli* cell quantification and live/dead estimates using the G2 counter

Visualizing an entire chamber at once is far easier (more user-friendly) and less prone to error than piecing the images together after imaging (as done with the G1 device). So, in the G2, trap size was conveniently set to be slightly smaller than our in-house microscope FoV (~ 180 µm, radius) at 1,000X magnification. This allows for one image for each chamber. However, FoV can be different for other microscopes. In G2, each chamber has a radius of 50 μm and 8 chambers of equal size (Fig. 3a). These 8 consecutive chambers can determine the chamber-to-chamber count variation and inconsistencies. High chamber variation gives rise to inaccurate total counts. We counted *E. coli* (1.8 × 10^8^ CFU/ml) cell numbers in G2 and PH counters. G2 showed statistically significant count variation among the average chamber counts in five replicates (average count between 20 and 35, *p* = 0.01) (Fig. 3c i).

The total count only varies by 8.6% (mean percent error) compared to the CFU/ml with no significant variation (*p* = 0.26). However, we observed a significant difference among the five replicates of G2 and PH count (*p* = 0.04), and PH and CFU/ml count (*p* = 0.03) (Fig. 3c ii). The PH count difference with the CFU/ml count was observed in the expected range (20%). The data indicates that smaller chamber size allows for the retention of fewer cells per chamber while maintaining consistency in chamber-to-chamber counts. These results also showed us the number of chambers to use for accurate counts; 8 chambers gave accurate results.

To further evaluate the capabilities of the G2 device, we estimated live and dead bacterial cells using live/dead fluorescence dyes PI and CFDA-SE. In this study, the *E. coli* cells were treated with 70% IPA for 1 h to produce dead bacterial cells and then stained with PI, a red fluorescence dye (Fig. 3d ii). In parallel, live cells were stained with the green fluorescence dye CFDA-SE (Fig. 3d iii). Red and green bacterial cells were confirmed using 1000× magnification imaging. The brightfield (BF) image of dead cells is shown in Fig. 3d i. Live/dead staining has been used in many studies such as cell viability assay, biofilm, and antibacterial resistance studies^70^, but it should be noted that these dyes come with their own errors and are only an estimation of live/dead bacteria^71,72^. This microfabricated counter can concurrently count and estimate live/dead cells.

### A novel G3 counter shows accuracy in counting and growing *E. coli* cells

Although we sufficiently improved counting accuracy in G2's design, we would like to improve the reliability of this counter because we observed chamber-to-chamber significant count differences (*p* = 0.01) (Fig. 3c i). We assumed that the chamber height (cell overlapping) had a significant impact on this inconsistency. Previously, a droplet-based microfluidics cell counter was studied with bacterial cells and showed that a specific concentration is needed to increase counting accuracy. They observed counting discrepancies when cells overlapped or were in close proximity^73^. Similarly, a multi-volume cell counter, focusing on mammalian cells, reported that the accuracy of cell counts is significantly impacted by chamber volume^1^. This is due to unequal distribution or the presence of too few or too many cells in the chamber. We considered these phenomena as well as our G2 findings when developing the G3 device by reducing the chamber height to 5 µm (Fig. 4a). Our goal is to count fewer cells in each chamber and minimize the overlapping cells while maintaining accuracy and reducing cell floating. The beauty of this system is that the design is very simple, and if needed, the chamber size can be changed, and more chambers can be added easily. Smaller chambers mean fewer counts per chamber, but adding more chambers leads to more overall counts per device. Thus, the modular design of the counter allows for easy adaptation for selected needs such as different microscopes FoVs.

To achieve high precision for imaging and counting, it is essential to have nearly immobile cells to maintain them in fewer phases. Initially, using five different concentrations of 1 μm beads (1.0 × 10^8^, 2.0 × 10^8^, 5.0 × 10^8^, 1.0 × 10^9^, and 2.0 × 10^9^ beads/ml), we empirically determined the optimal number of counts per chamber that showed reduced fluctuation compared to the supplier count. We observed a strong correlation between expected and G3 counts (R^2^ = 0.98) (Fig. 4c i). As expected, we saw that while concentrations are increasing, the cell numbers are likewise increasing, resulting in a strong correlation (R^2^ = 0.998) (Supplementary Fig. S3i). The total count showed a 5.4% and 22% difference with concentrations of 2.0 × 10^8^ beads/ml and 1.0 × 10^8^ beads/ml, compared to the expected counts (Fig. 4c ii). However, at higher concentrations (2.0 × 10^9^ beads/ml), a large number of beads in the chamber caused a significant count difference (Fig. 4c ii and Supplementary Fig. S3ii). These results demonstrate that an optimal range of bead numbers is required to enhance count accuracy. Too many or too few beads in the chamber can lead to count variation. We found that average bead numbers ≤ 25 can maintain less count variation.

However, our goal is to count small bacteria cells. Based on the optimized concentration with 1 µm beads, we then counted *E. coli* (1.8 × 10^8^ CFU/ml) using G3. We observed no significant inconsistency in the chamber-to-chamber count (*p* = 0.58) (Fig. 4d i), which made it more reliable than G2 (*p* = 0.01) (Fig. 3c i). This new design reduced the count difference by 7.9% with no significant difference (*p* = 0.22) compared to CFU/ml (Fig. 4d ii). G3 is more accurate than G2. Additionally, we tested the count variation between G3 and PH counters, which showed no significant difference among the chamber counts (*p* = 0.55), but PH and CFU/ml showed significant count differences (*p* = 0.03) (Fig. 4d ii). We also tested lower and higher concentrations of cells than the optimized range (1.0 × 10^8^, 2.0 × 10^8^, 5.0 × 10^8^, 1.0 × 10^9^, and 2.0 × 10^9^ beads/ml), as expected, we observed a huge count variation as demonstrated in 1 µm bead count (Fig. 4c ii). A droplet-based microfluidic study reported that a high concentration of *E. coli* cells mostly occupied in a droplet caused counting discrepancies^73^. Our results demonstrate that the G3 design accurately counts *E. coli* cells. However, G3 has several features that the PH counter lacks. The G3 simplifies image processing due to lower depth, eliminating the need for capturing Z-stack images, a requirement for an accurate PH count (without the Z-stack, PH counts can be over- or under-estimated). The G3's chamber size aligns with the microscope's field of view, streamlining imaging and counting processes. In contrast, PH counters necessitate multiple images, especially for small bacteria, requiring meticulous efforts in image acquisition, merging, and counting. The G3 device consistently maintained cell capacity and counting accuracy (compared to CFU/ml) and can be used for real-time growth assays after counting bacterial cells that lack in PH counter. Existing counting methods (e.g. flow cytometry, hemocytometer, etc.) are not often used to observe real-time population growth after counting. This might be a constraint for studies that require contemporaneous data. As such, we developed a device that can monitor bacterial growth within a cell counter. *E. coli* cells can grow inside the G3 chamber using LB media; see a time-lapse movie showing growth at 0 h, 3 h, 6 h, and 12 h (Fig. 4e, Supplementary Movie S3). This demonstrated that further improvement of this counter would lead to real-time observation of bacterial dynamics at the single-cell level.

Our data suggests that variations in cell counting at high and low concentrations can be minimized through an improved counter design, such as increasing the number of chambers, reducing the size of additional channels, and shortening chamber-to-chamber distance. In our current design, we have incorporated several features, including a reservoir to manage initial flow turbulence, connection channels, and designated inlet and outlet zones for loading and releasing samples. However, we observed that, at times, a number of cells or beads become trapped in various zones, potentially leading to counting inaccuracies or variations. To minimize cells sticking to PDMS we washed the device with Tween-20 (a non-ionic surfactant that prevents cell adhesion to the PDMS surface^74,75^). Additionally, the entire counter was observed via microscopy, and the number of beads/cells in each chamber was compared. It is worth noting that common practice in microfluidic device design involves incorporating additional channels, traps, or chambers, as exemplified by the use of slope channels in multi-volume hemocytometers to prevent air bubble trapping^1^. Notably, the objective of this study is to optimize the technique to produce a more efficient system through subsequent improvements.

## Conclusion

We developed three sequential (G1, G2, and G3) counter-on-chip devices while optimizing the 2PP fabrication and PDMS chip construction procedures. Using the G1 counter, we demonstrated the impact of chamber size on counting accuracy by increasing the chamber volume by a factor of two. The larger chamber volume or size of G1 had little negative effect on counting accuracy. Because G1 posed challenges in imaging and data processing, we developed G2 with a smaller chamber size, showing minimal counting discrepancies within each chamber. We then developed G3 by reducing the depth, and this helped keep bacteria in fewer phases due to reduced cell floating; the cells quickly became immobile, allowing for counting and estimation of live/dead cells.

Our detailed procedures simplify the process of constructing structures using the 2PP system and producing PDMS-based chips. This will help others to develop their own microfluidic chips quickly and efficiently. Our group previously had great success designing and using custom microfluidic devices produced by the traditional photolithography approach^76,77^; however, we see 3D printing as the future for microchemostatic bacterial microfluidic devices. However, two major limitations of using 3D printers for bacterial study have been the resolution needed to print small structures for small organisms (the new 2PP system has overcome these limitations), and a straightforward method of doing so quickly and inexpressively. We describe this here while producing useful and novel devices.

Studying and quantifying persisters or testing for the existence of VBNC (viable but nonculturable) is challenging. These experiments require precise counts. It is important to note that using our devices and PH as the manufacturer described, we see no evidence of VBNCs in log phase (compared to CFU/ml) in our strain of *E. coli*. This is in direct contrast to previous reports of VBNCs in this phase with a very closely related strain of *E. coli*^35–37^. The original identification of VBNCs might be the result of count discrepancy between CFU/ml and hemocytometer. The error may come from hectic Z-stack imaging and floating cells. However, our G3 device was designed not to require Z-stacks, which reduces the likelihood of overestimation of VBNCs. Further modifications to this system may help in addressing the challenges in bacterial studies.

## Materials and methods

### Counter-on-chip design and post-processing

Microfluidic counters (G1, G2, and G3) were designed using Autodesk Fusion 360, a 3D CAD (Computer Aided Design) software. The G1 counter comprised six round chambers, including three counting chambers, with radii of 500 µm, 700 µm, and 1000 µm, respectively, an inlet and outlet (to add and remove samples from the device) with radii of 1000 µm, and one reservoir with a radius (r) of 1400 µm. The depth of G1 was set at 20 µm, similar to the depth of the PH counter (Fig. 2b ii). The inlet was used to load the sample that flows through the reservoir and release the remaining sample to the outlet. Furthermore, we designed second and third generation G2 and G3 counters, consisting of eight counting chambers of equal size (r = 50 µm) along with an inlet, outlet, and reservoir with depths of 20 µm and 5 µm, respectively. The specific dimensions of these counters are depicted in Figs. 3a and 4a. The CAD files of the structures were exported to stereolithography (.STL) format and then imported into DeScribe (v2.6) (Slicing software, Nanoscribe GmbH) to generate a 3D print general writing language (.GWL) file and then sent to NanoWrite (v1.10) (Nanoscribe GmbH) for 3D printing.

### The 2-photon polymerization fabrication process

#### Materials selection, substrate preparation, and increasing adhesion

Nanoscribe GmbH manufactured silicon substrate (SS) (square: 25 × 25 × 0.7mm^3^, RI 3.7 @780 nm), multi-DiLL sample holder, high viscous negative-tone IP-S photoresist, and 25 × NA0.8 immersion objective (with felt ring) were used for high-resolution 3D printing. The hydrophobicity and adhesion properties of the SS were improved by a modified silanization process (Fig. 1a). Initially, the SS was dried at 90 °C for 10 min. and then rinsed with pure acetone, followed by isopropanol and dH_2_O; SS incubated for 15 min. at room temperature, and then dried with nitrogen (N_2_) or air. Next, SS was cleaned with UVO_3_-cleaner (Jelight, Model 24) for 3 min. and soaked in 3-(Trimethoxysilyl)-propyl methacrylate (TMSPM) ethanol solution (0.5% *v/v*) (TCI Chemicals, USA) for 12 h. Lastly, the SS was rinsed with pure acetone, then dH2O, and then dried with N_2_/air.

#### 3D Printing process

The structure fabrication was performed using an ultra-precision 2PP system (Photonic Professional GT2, Nanoscribe GmbH). The system uses a 780 nm-pulsed femtosecond fiber laser, allowing high-resolution microstructure printing with a short manufacturing time. DeScribe (v2.6) (Slicing software, Nanoscribe GmbH) was used to generate a specific job file. The parameters for this file included the process recipe: IP-S 25 × ITO Solid (3D MF), Slicing Mode: Fixed, Fill Mode: Solid (hatching distance 0.5 µm), scan mode: Galvo, 100 mm s^-1^ writing speed, and Configuration: Dip-in Laser Lithography. However, silicon has a very high refractive index (RI 3.7 @780 nm); thus, printing on it requires reconfiguring the system’s ‘Defined Focus’ setting. The job file was then uploaded into NanoWrite (v1.10) (Nanoscribe GmbH) for the printing process. A single drop of resin was applied using a lid spatula. DeScribe showed the required volume of resins before processing. This confirmed that one drop of resin met the specified requirements. The printing duration for the G1, G2, and G3 counters was approximately 3.4 h, 1.4 h, and 0.45 h, respectively.

#### Sample development and surface coating

Under the fume hood, the polymerized SS was submerged vertically using a dedicated holder in a glass beaker containing propylene glycol monomethyl ether acetate (PGMEA, Spectrum Chemical) for 30 min. to dissolve the unpolymerized resin. To remove the excess PGMEA, SS was immediately immersed in pure isopropanol for 5 min. and then gently dried using a ball blower. The SS was then placed in a vacuum chamber with 10 µL of 1H, 1H, 2H, 2H-Perfluorooctyltriethoxysilane (FOTS) (Alfa Aesa, USA) for 1 h, followed by isopropanol rinsing, N_2_/air drying and baked at 90 °C for 30 min. (Fig. 1b). This process is referred to as flurosilanization. FOTS can modify the silicon substrate’s surface super-hydrophobic and act as an anti-adhesive layer to remove the cured PDMS from SS easily.

### SEM and SEM–EDS imaging

The flurosilanized SS, here referred to as master mold (MM). MMs were coated with a thin layer (15 nm) of gold (Au) using a sputter coater system (CrC-150, Plasma Science). Next, the MM was characterized using a scanning electron microscopy (SEM) imaging system (Hitachi-S3400N, Japan). An SEM-energy dispersive X-ray spectroscopy (SEM–EDS) (Hitachi-S4700, Japan) was performed to identify the chemical composition of the MM. The EDS was conducted with the flat structure, and quantitative analysis was done using AZtec software. SEM image acquisition was performed with an acceleration voltage of 10 kV (for G1) and 5 kV (for G2 and G3); however, 20 kV was used for SEM–EDS. SEM images were acquired with a 30^0^ tilt angle to validate the thickness of the MM.

### PDMS chips construction

For replica molding, PDMS (Sylgard 184, Dow Corning) was prepared using a prepolymer and cross-linker at a 10:1 (w/w) ratio^78^. Then, PDMS was poured over the MM and degassed using a vacuum desiccator for 30 min. to remove the air bubbles and then cured at 90 °C for 24 h. Next, the PDMS replica was carefully peeled off, and the MM was preserved for future use. Next, the inlet and outlet of the PDMS replica were drilled using a biopsy puncher (1 mm, Harris Uni-Core). The PDMS replica was degassed for 20 min. followed by 1 h sonication (Branson 1800 cleaner) using pure methanol to remove residues or contaminants in the PDMS channel. Then, the PDMS replica was gently dried with N_2_/air and cured at 90 °C for 20 min. This prepares the PDMS mold for glass bonding (Fig. 1b). Glass surface activation is required for efficient glass-PDMS bonding. Firstly, cover glasses (corning, 24 × 40 mm, thickness: 0.16—0.19 mm) were soaked in 70% H_2_SO_4_ for 24 h, rinsed with dH_2_O, dried with N_2_/air, and stored in pure (99.5%) methanol for 24 h. Next, we washed and soaked the stored glass in pure (99.5%) methanol overnight. Before bonding, the glass slide was cleaned using pure acetone and isopropanol, dried with N_2_/air, and kept at 90 °C for 15 min. Acetone cleans organic residues, and isopropanol cleans contaminated acetone residues. Finally, the cover glass and PDMS mold were placed in a UVO_3_-cleaner (Jelight, Model 24) for 3 min. Subsequently, the PDMS mold was gently placed on the cover glass and kept at 90 °C for 24 h (Fig. 1b). At this point, the PDMS chip is ready for use or can be stored in a covered Petri plate.

### Microbeads and culture preparation

Fluorescent polystyrene microbeads (1.0 µm, blue-green, ThermoFisher, USA) were suspended into 2% Tween-20 (VWR-chemicals, USA) at a concentration of 1.0 × 10^10^ beads⁄ml. Similarly, Carboxylated PS microspheres (5.0 µm, yellow-green, Magsphere, USA) were suspended into 2% Tween-20 at a concentration of 3.4 × 10^8^ beads/ml.

*Escherichia coli* (*E. coli)* DH5αZ1 was used in this study. An overnight culture was grown in Miller’s lysogeny broth (LB) media at 37 °C and shaken at 300 rpm until it reached the mid-exponential phase (~ OD 0.5). The colony forming units (CFU/ml) method was used to count the bacteria on traditional agar plates. Plates were incubated at 37ºC for 48 h, and then scanned on a flatbed scanner. Custom Python scripts were used to identify and count bacterial colonies^13,79^. For live cell visualization, *E. coli* cells were stained with a green fluorescent CFDA-SE (5-(and-6-)-carboxyfluorescein diacetate, succinimidyl ester) (ThermoFisher, USA) dye at a final concentration of 10 µM. To visualize dead *E. coli* cells, a red fluorescent propidium iodide (PI) (Sigma, USA) dye was used at a final concentration of 1 µl/ml. The staining procedure followed the manufacturer's instructions.

### Microfluidic device assembly, sample run, and image acquisition

The microfluidic PDMS chip was affixed to a stable, non-drifting flat base and positioned beneath an inverted microscope (Nikon Eclipse Ti2-E). The use of a flat surface ensures a seamless flow of liquid and enables control of the flow direction. Initially, 0.5 mL 2% Tween-20 was used to flush the device from the inlet to the outlet. Tween-20, a non-ionic surfactant, serves the purpose of preventing cell adhesion to the PDMS surface^74,75^. Notably, the round-shaped microfluidic counter facilitates smooth particle/cell movement without sticking at the device edges.

A 1 ml syringe was used to load 0.2 ml of sample (e.g., 1 µm, 5 µm beads, and *E. coli* cells) into the inlet. After confirming the smooth flow of the sample through the outlet, the microfluidic devices (e.g., G1/G2/G3) were gently placed under the microscope. After about 20–25 min. the flow rate stabilized inside the chamber (Supplementary Movies S1 and S2). Image acquisition of the G1, G2, and G3 devices was conducted using an inverted microscope (Nikon Eclipse Ti2-E) coupled with 10x (Nikon, N.A. 0.45) and 100x (oil immersion N.A. 1.45) objectives, along with NIS element AR software. This advanced microscopy system has a custom microscope lexan enclosure that can control temperature and humidity. Nikon Sola SE II light engine (365 nm) was used for taking fluorescence images. The temperature was set to 37 °C during both the experiment and imaging of microbeads and *E. coli*. The Petroff-Hausser counter (Hausser Scientific) was used for estimating microbeads and *E. coli* cell numbers. The standard operating procedure was followed per the manufacturer’s manual for sample preparation and quantification of microbead and *E. coli* concentrations. An open-source software, Fiji ImageJ^80^, and custom Python script^13^ were used for image analysis and quantification of microbeads and bacterial cells. To validate the accuracy of our automated program, manual cell counting was periodically performed. A detailed experimental procedure and image processing are described in Supplementary Fig. S1i–iii.

### Statistical analyses

OriginPro version 2023b (OriginLab Corporation, USA), Microsoft Excel, and a web-based R programming application^81^ were employed for statistical data analysis and plotting. Statistical significance was determined using a one-way ANOVA (analysis of variance) followed by post hoc tests (multiple comparisons); an f-test to determine variance (*p* < 0.05 was considered to have significant variance), followed by a two-tailed t-test with unequal variances (if F statistic > F critical value) or equal variances (if F statistic < F critical value).

### Calculation

The combination math approach (Fig. 2c ii, d ii) was determined using the following equation:

$$^{n} C_{r} = \frac{n!}{{r!\left( {n - r} \right)!}}$$

here n = number of replicates, and *r* = number of random selections (ranging from 3 to 8). Using the above equation, we determined the combinations of randomly selected replicates. For example, when n = 8, and *r* = 3 (referred to as a 3-rule replicate), we obtained a total of 56 distinct values (replicates). Subsequently, we computed the total counts for all these 56 replicates. Similarly, we calculated the total count for 4–8 rule replicates. This method allowed us to systematically expand the number of replicates, facilitating a more robust assessment of the coefficient of variation (CV).

For G1, G2, and G3 counter-on-chip, the total count of microbeads (1 µm and 5 µm) and *E. coli* cells was calculated using the following equation:

$${\text{Concentration }}\left( {\frac{{{\text{beads}}/{\text{cells}}}}{{{\text{mL}}}}} \right) = \frac{{{\text{ Number}}\,\,{\text{of }}\,{\text{beads}}/{\text{cells}} \times 1000}}{{{\text{chamber }}\,\,{\text{volume}},{ }\,\,{\mu L}}}$$

In the main text, we mentioned that the count varies by specific percentage, which means that the total count is either lower or higher compared to the expected counts. This is because individual replicates have different percent error, either lower or higher numbers. We calculated the Mean Percent Error (MPE) from the expected count. This is a commonly used method for calculating percentage error in microfluidic devices^82–90^.This method helps quantify the overall trend of variations across multiple replicates, whether they are consistently higher or lower than expected. The resulting MPE provides a measure of the average magnitude and direction of the deviations from the expected counts. MPE was calculated using the following equation:

$${\text{MPE}} = { }\frac{{Estimated_{mean} - Expected_{mean} }}{{Estimated_{mean} }} \times 100\%$$

where $Estimated_{mean}$ = average number of estimated or observed counts of cells/beads for the *i*-th replicates; $Expected_{mean}$ = reference or expected mean for the i-th replicates.

### Supplementary Information

Supplementary Video 1.

Supplementary Video 2.

Supplementary Video 3.

Supplementary Information 1.

## Data Availability

All the data that supports the findings of this study are included in this published article (and its supplementary information files). Also, any additional data is available from the corresponding author upon request (email: nicholas.butzin@gmail.com).
