# Supplementary material for: Counter-on-chip for bacterial cell quantification, growth, and live-dead estimations
Source: Sci Rep. 2024 Jan 8;14:782. doi: 10.1038/s41598-023-51014-2 (PMC10774380; doi:10.1038/s41598-023-51014-2)
Supplement: Supplementary file 4 — Supplementary Information 1. [file 41598_2023_51014_MOESM4_ESM.pdf]

Counter-on-chip for bacterial cell quantification, growth, and live-dead estimations

K M Taufiqur Rahman[1] and Nicholas C. Butzin[1,2*]

[1]Department of Biology and Microbiology; South Dakota State University; Brookings, SD, 57006; USA.
[2]Department of Chemistry and Biochemistry; South Dakota State University; Brookings, SD, 57006; USA.

*Correspondence: nicholas.butzin@gmail.com

Supplementary Information

Microfluidic Device assembly, sample run, and microscopy imaging:

Step 1: The PDMS-based counter-on-chip was attached to a non-drifting flat base and set under an inverted microscope (Fig. S1 *i*). A flat surface ensures smooth liquid flow and flow control from one direction to the other.

Step 2: Initially, 0.5 mL 2% Tween-20 was used to flush the device from the inlet to the outlet. Tween-20 is a non-ionic surfactant that helps to prevent cell/particle adhesion to the PDMS surface[1, 2]. Notably, the round-shaped microfluidic counter helps smooth particle/cell movement without sticking at the edge.

Step 3: Next, at least 0.2 mL of samples were loaded from the inlet using a 1 mL syringe (Fig. S1 *i*). The particles/cells stopped moving around ~20-25 mins (supplementary movies S1 and S2). Fig. S1 *ii* shows one chamber of the G1 with 5 µm microbeads.
For counting 1 µm microbeads and *E. coli* cells (with G2 and G3) similar procedure (Steps 1-3) was applied. Microscopic imaging was taken with 100x magnification for G1, and 1000x for G2 and G3, as *E. coli* are relatively small, which needed higher magnification for precise imaging and subsequently counting.

Step 4: After the imaging was done, we initially conducted cell/bead quantification through manual counting, which was quite time-consuming and not user-friendly. Fiji ImageJ[3] is a very well-known image analysis software. Using this tool, we counted cell/bead and compared the results with manually counting data. We found an exact match. An example of the counted masked image is shown in Fig. S1 *iii*.
For a small number of images this tool can be very handy. However, a large number of images requires additional expertise. In this case Fiji ImageJ macros record command could be an option[3], but specific programming skill is required to get this done efficiently. We wanted to keep this counting simple, so we used our previously developed image-processing method that uses custom computer code and leverages machine-learning algorithms[4, 7-9].
The code will be found at https://github.com/hdeter/CountColonies.

Step. 5: To validate counting results, we performed random checks on images through manual counting, which showed a consistently low error rate, often falling below 1%. This underscores the accuracy and reliability of our counting methodology.

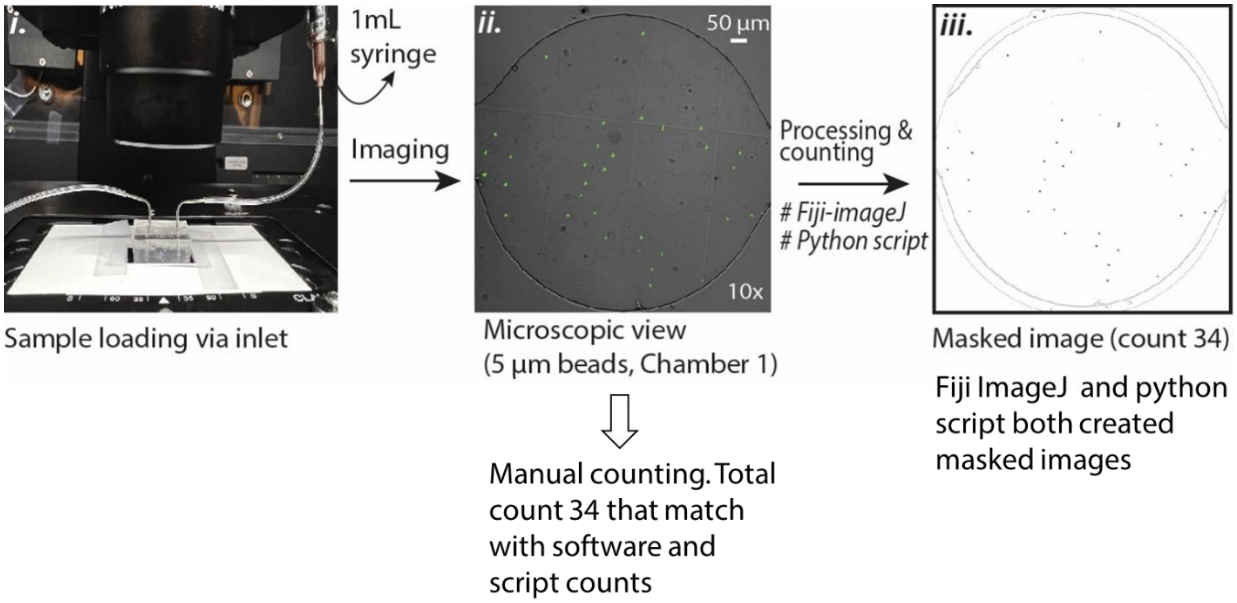

Fig. S1: G1 counter setup, imaging, and data analysis: **(i)** Microfluidic counter-on-chip under an inverted microscope. The microfluidic holder (white flat base) was printed using Fused Deposition Modeling (FDM) 3D printer; material used: biodegradable thermoplastic polyester known as polylactic acid (PLA). **(ii)** As an example, we showed chamber 1 of the G1 counter with 5 µm microbeads; 100x magnification, scale bar: 50 µm. **(iii)** The masked image showed precise counting.

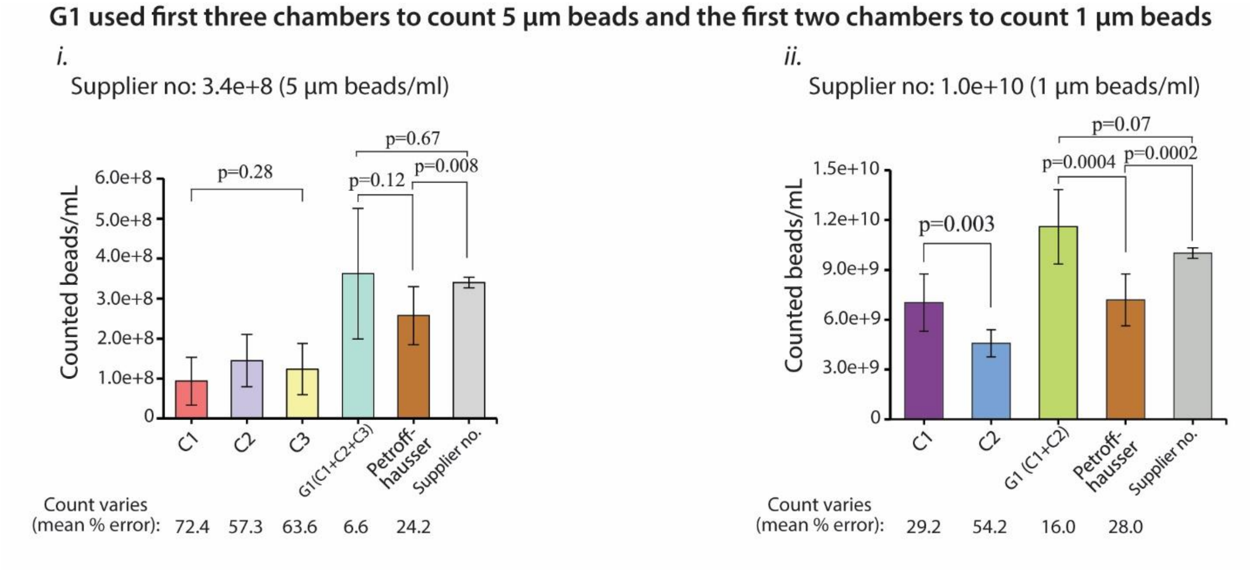

Fig. S2: The G1 device showed counting accuracy with two different size beads. **(i)** For 5 µm beads, there was no statistically significant variation was observed among the three different chambers ($p = 0.28$, $n = 8$). The individual total count in each chamber (C1-C3) showed a significant count difference (C1 = 72.4%, C2 = 57.3%, and C3 = 63.6%) compared to the supplier counts. However, G1 (C1+C2+C3) showed less count discrepancy (6.6%) with no statistically significant variation ($p = 0.12$, $n = 8$) compared to Petroff-Hausser (PH) count. PH showed a count variation of 24.2% compared to supplier count (also see Fig. 2 c *i*). **(ii)** 1 µm

beads were counted using two chambers (C1 and C2). As expected, the total count difference was found to be statistically significant ($p = 0.003$, $n = 8$). The C1 chamber count was varied by 29.2% and, C2 by 54.2% compared to the supplier counts. However, the combination of C1 and C2 chamber counts showed a 16% difference compared to the supplier count, with statistically significant count variation ($p = 0.0004$, $n = 8$). We observed these significant chamber-to-chamber count differences because the chamber volume increased two-fold (see Fig. 2a). n: Number of replicates.

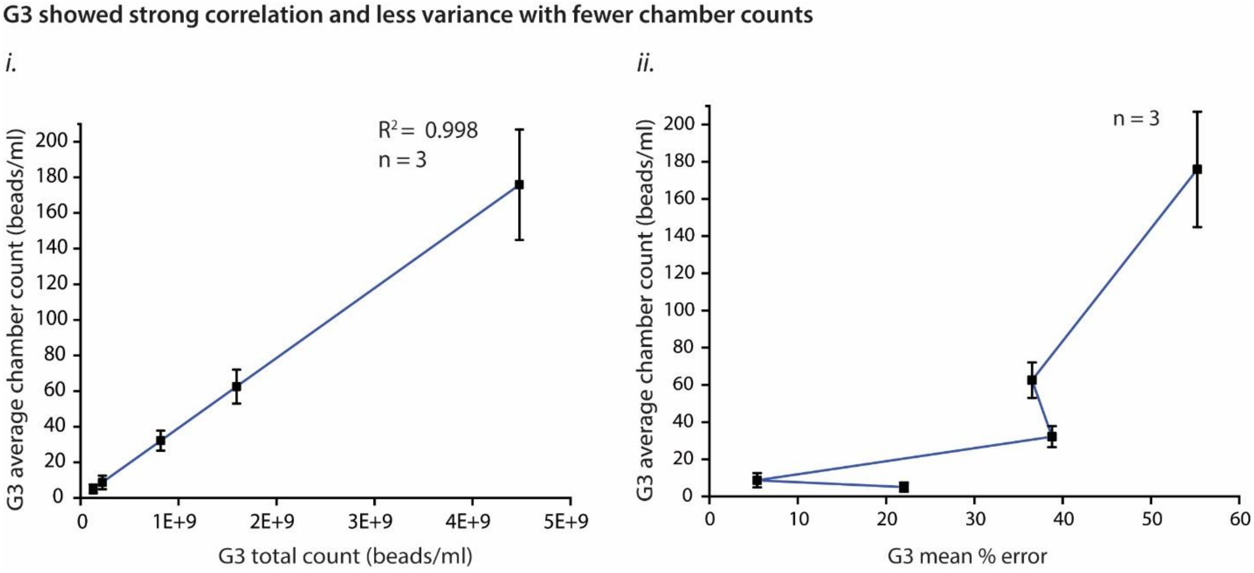

Fig. S3: G3 maintained a high correlation and reduced count variation. **(i)** Strong correlation was observed between the average chamber count and total count in G3 ($R^2 = 0.998$, $n = 3$). n: Number of replicates. This suggests that the retention capacity of G3 is not random; it increases when the concentration is increased. However, it's essential to note that a minimum concentration is required to achieve accurate counts. **(ii)** The accuracy of counts in the G3 chamber is enhanced when a minimum number of microbeads are present. For instance, when the average chamber count is lower, the total count difference is smaller. Conversely, when the average chamber count is higher, the count difference is dramatically increased compared to the supplier count. This underscores the importance of maintaining an optimal concentration in the chamber. Similar to the other systems, our G3 showed accuracy in counting with an optimum range of concentrations.

Supplementary References:

1. Wu, M.H., *Simple poly (dimethylsiloxane) surface modification to control cell adhesion.* Surface and Interface Analysis: An International Journal devoted to the development and application of techniques for the analysis of surfaces, interfaces and thin films, 2009. **41**(1): p. 11-16.
2. Liu, V.A., W.E. Jastromb, and S.N. Bhatia, *Engineering protein and cell adhesivity using PEO-terminated triblock polymers.* Journal of biomedical materials research, 2002. **60**(1): p. 126-134.
3. Schindelin, J., et al., *Fiji: an open-source platform for biological-image analysis.* Nature methods, 2012. **9**(7): p. 676-682.
4. Deter, H.S., et al., *A cell segmentation/tracking tool based on machine learning*, in *Computer Optimized Microscopy*. 2019, Springer. p. 399-422.
5. Deter, H.S., et al., *Proteolytic queues at ClpXP increase antibiotic tolerance.* ACS synthetic biology, 2019. **9**(1): p. 95-103.
6. Hossain, T., et al., *Antibiotic tolerance, persistence, and resistance of the evolved minimal cell, Mycoplasma mycoides JCVI-Syn3B.* Iscience, 2021. **24**(5): p. 102391.
7. Butzin, N.C., et al., *Marching along to an offbeat drum: entrainment of synthetic gene oscillators by a noisy stimulus.* ACS synthetic biology, 2016. **5**(2): p. 146-153.
8. Butzin, N.C., et al., *Entrainment of a bacterial synthetic gene oscillator through proteolytic queueing.* ACS synthetic biology, 2017. **6**(3): p. 455-462.
9. Butzin, N.C. and W.H. Mather, *Crosstalk between diverse synthetic protein degradation tags in Escherichia coli.* ACS synthetic biology, 2018. **7**(1): p. 54-62.
